# Regulation of microglial physiology by the microbiota

**DOI:** 10.1080/19490976.2022.2125739

**Published:** 2022-09-24

**Authors:** James Cook, Marco Prinz

**Affiliations:** a Institute of Neuropathology, Medical Faculty, University of Freiburg, Freiburg, Germany; b Centre for NeuroModulation (Neuromodbasics), University of Freiburg, Freiburg, Germany; c Signalling Research Centres BIOSS and CIBSS, University of Freiburg, Freiburg, Germany

**Keywords:** Microglia, development, microbiota, neurological disorders, gut-derived metabolites, gut-brain axis

## Abstract

The mammalian gut contains a large, complex community of microorganisms collectively termed the microbiota. It is increasingly appreciated that gut microbes are closely integrated into mammalian physiology, participating in metabolic symbiosis, promoting immune function and signaling to a wide variety of distant cells, including the brain, via circulating metabolites. Recent advances indicate that microglia, the brain’s resident immune cells, are influenced by microbial metabolites at all stages of life, under both physiological and pathological conditions. The pathways by which microbiota regulate microglial function are therefore of interest for investigating links between neurological disorders and gut microbiome changes. In this review, we discuss the effects and mechanisms of microbiota-microglia signaling in steady state, as well as evidence for the involvement of this signaling axis in CNS pathologies.

## Introduction

It is increasingly appreciated that mammalian physiology depends not only on the host genome, but also by intimate interactions with commensal microbes which have been shaped by millions of years of co-evolution. Some such interactions are mutualistic, such as co-operative food digestion and nutrient synthesis, though some aspects of physiology are influenced by the mere presence of commensals or by infection by pathogenic species. Thus, the physiology of most animals can be best understood as a meta-organism comprising the host and a huge diversity of commensal microorganisms collectively referred to as the microbiota. Host-microbiota interactions are increasingly understood to be fundamental in shaping diverse aspects of mammalian biology, including development, metabolism, immunity and neurological function. The link between intestinal microbiota and brain function has been extensively described, and is commonly referred to as the microbiota-gut-brain axis.^1^

Microglia are one of the four primary cell types comprising the mammalian central nervous system (CNS). As the brain’s primary immune population, they contribute not only to the development and maintenance of the CNS, but also critically regulate CNS disease states such as neurodegeneration, autoimmunity and neurodevelopmental disorders.^2^ Although separated from commensal microbes by both the gut barrier and blood–brain barrier, the presence of intestinal microbes has been demonstrated to regulate microglial phenotype and functions, both at steady state and under disease conditions. Given the increasing interest in the gut microbiome as a regulator of neurological disorders, direct communication between intestinal microbiota and microglia is an intriguing concept which may prove to be a central mechanism linking the microbiome to various brain diseases. In this review, we explore recent advances in understanding the mechanistic links between gut flora and microglia, as well as their consequences for neurological disorders.

### Microglia and brain development

**Figure 1.**
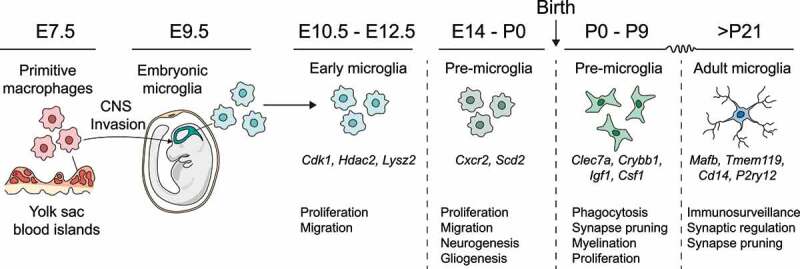
Microglial development. Microglia emerge from yolk-sac precursors and colonize the developing brain at around E9.5. Subsequently, they undergo a series of stepwise transformations, acquiring diverse developmental phenotypes as pre-microglia before maturing into adult microglia during the second or third postnatal week.

Microglia are the brain’s tissue-resident macrophages, comprising between 5% and 15% of all cells in the healthy brain. Microglia originate as primitive c-kit^+^ macrophages in the embryonic yolk sac, which proceed to colonize the embryonic brain at around E9.5, quickly spreading to all developing brain structures.^3–6^ Due to the establishment of the blood–brain barrier at E13.5, the brain is shielded from subsequent waves of hematopoiesis originating in the fetal liver and bone marrow. Thus, there is no replacement of yolk-sac-derived microglia with monocyte-derived cells under homeostatic conditions,^7^ as is observed in other tissues.^8^ Instead, microglia self-renew throughout life via tightly regulated local proliferation and apoptosis.^9,10^ After first colonizing the brain at E9.5, microglia undergo several stepwise maturation stages (Figure 1).^11,12^ The first major transition involves a switch from early to pre-microglia at around E14.5.^11,12^ Pre-microglia then gradually acquire adult-like properties after birth, maturing fully into adult microglia beginning around the second or third postnatal week.^11,12^ Adult microglia exhibit a stereotypical, relatively homogenous signature enriched for genes involved in immunosurveillance, including the purinergic receptors *P2ry12* and *P2ry13*, as well as *Trem2* and *Cd14*.^11,13^ By contrast, earlier developmental ages are marked by far greater microglial diversity, with distinct microglial subsets exhibiting spatiotemporally regulated signatures and functions.^13–15^

The timing of microglial entry into the brain means they are well positioned to influence CNS development. For instance, in the early postnatal brain microglia provide critical trophic support to oligodendrocyte precursor cells in the corpus callosum and cerebellar white matter, and loss of microglia during this time leads to later defects in myelination.^16,17^ Microglia also influence neuronal development, with some neuron subsets reliant on microglia for trophic signaling and correct positioning.^18^ Additionally, microglia are known to engulf apoptotic cells throughout development and adulthood, which is critical to tissue homeostasis. Interestingly, microglia are also capable of inducing developmental death of otherwise viable cells via direct engulfment, in a process termed “phagoptosis”.^19^ Through this process, microglia can directly fine-tune cell numbers during CNS development, including the extent of neurogenesis.^20,21^

Perhaps the best-studied function of microglia in neurodevelopment is complement-dependent synapse elimination, whereby superfluous synapses are tagged sequentially by C1q and C3, leading to their engulfment by CR3-expressing microglia.^22–24^ Loss of complement components or depletion of microglia therefore inhibits developmental circuit refinement, resulting in functional connectivity deficits in adults.^25,26^ Interestingly, aberrant synaptic refinement is postulated to be a central mechanism governing some psychiatric disorders, in particular schizophrenia, which has been linked to numerous polymorphisms affecting immune receptors including the complement system.^27^ Moreover, various neurodegenerative pathologies including Alzheimer’s disease have been suggested to encompass some degree of aberrant synaptic elimination by microglia.^28^ Thus, microglia are capable of directly shaping CNS architecture through a variety of mechanisms. Tight regulation of these processes is important for optimal CNS function, and disturbances to microglia during critical developmental windows can affect the course of brain maturation and susceptibility to neuropsychiatric and neurodevelopmental disorders.^26,29^ Similarly, microglial dysfunction in aging may play a key role in neurodegeneration and cognitive impairment

### Microglia in CNS homeostasis and disease

Adult microglia exhibit several marked adaptations to their role as the primary immunocompetent cells of the brain. Firstly, their unique transcriptomic signature encompasses a large repertoire of genes involved in immunosurveillance.^30^ These include pattern recognition receptors such as toll-like receptors (TLRs), scavenger receptors and triggering receptor expressed on myeloid cells 2 (TREM2). Moreover, mature microglia express the receptors P2RY12 and P2RY13, conferring exquisite sensitivity to purinergic stimuli. Secondly, their long, elaborately branched processes are remarkably motile, undergoing extremely dynamic, rapid rearrangements and making contact with surrounding CNS cells on a constant basis.^31,32^ Thus, via their extensive sensome and unceasing motility, microglia scan the entire CNS space once every few hours, and are the primary responders to any disturbance in CNS homeostasis.

Following CNS insults such as injury, infection and neurodegeneration, microglia sense local cues and undergo transformation to reactive phenotypes. In general, these reactive microglia downregulate some homeostatic markers such as *P2ry12, Tmem119* and *Mafb*, and induce genes involved in effector functions, many of which vary depending on the specific insult. Recent studies support the existence of multiple states of reactive microglia which can be induced by different pathologies such as neurodegeneration and autoimmunity and engage diverse reactive functions including phagocytosis, proliferation, antigen presentation and inflammatory cytokine secretion.^13,14,33–35^ Whilst microglial responses are undoubtedly critical for effective resolution of insults and preventing damage to the CNS, there is ample evidence to suggest that inappropriate microglial activity can be a causative factor in various CNS diseases.^35^ Microglia are therefore a key candidate for transducing environmental factors such as microbiome-related signaling to the exacerbation or amelioration of CNS pathology.

## Microbiome-microglia interactions

The gut microbiota are known to exert extensive effects on host physiology via a variety of mechanisms. The most prominent mutualistic function of gut bacteria is metabolism – collectively the bacteria inhabiting the gastrointestinal tract contain many more metabolic genes than humans possess, thus allowing much greater metabolic flexibility than would otherwise be possible.^36^ In this sense, the gut microbiome is often described as an additional metabolic organ, assisting with fiber fermentation, tryptophan metabolism and detoxification of xenobiotics, amongst a wide range of other pathways. However, the effect of bacterial metabolism on mammalian physiology goes far beyond simple nutrient provision. Hundreds of bioactive metabolites are produced by the microbiota, circulating throughout every organ and affecting the activity of huge numbers of target cells.^37–39^

Of particular relevance to the CNS is the concept of the gut-brain axis, which describes numerous pathways linking gut physiology to brain function.^1^ The microbiota are critical players in the gut-brain axis, and can affect CNS physiology by directly modulating neurotransmission and neurodevelopment, by signaling through the vagus nerve, as well as by modifying endogenous metabolism, endocrine signaling and immune system activity (Figure 1).^1,40,41^ Studies in germ-free mice raised without any exposure to microbes, as well as in mice which have undergone microbiota depletion via broad-spectrum antibiotics, have revealed striking dependence of mammalian physiology on microbially derived cues. It is now increasingly appreciated that microglia are important target cells in the microbiota-gut-brain axis and may be critical in transducing changes in microbiome composition to altered brain function.

### Microbiota modulate microglia in development and homeostasis

Microglial phenotypes are instructed by the microbiota throughout life, even prior to birth (Figure 1). Recent data indicate that microglia from embryos of germ-free mice exhibit large differences when compared to those of SPF embryos at E18.5.^12^ This effect was reported to be highly sex-dependent, with male embryos displaying far greater numbers of differentially expressed genes than females.^12^ Lack of a maternal microbiome resulted in greater microglial density in the cortex at E14.5 and E16.5, which persisted to E18.5 in male embryos. By contrast, the transcriptional effect of the microbiota on male mice was diminished postnatally, with females displaying much greater transcriptomic differences between germ-free and SPF microglia at P20.^12^ These findings add to a growing body of evidence that fetal neurodevelopment is influenced by cues derived from the maternal microbiota and place microglia as key players in mediating their effects.

Interactions between microglia and colonizing commensals are also important for early postnatal neurodevelopment.^42^ Germ-free pups exhibit defective synaptic pruning in Purkinje cells, which is linked to decreased numbers of amoeboid microglia and aberrantly low expression of CD45, CD68 and scavenger receptors.^42^ Intriguingly, both microglial phenotype and synaptic pruning can be rescued by colonization of GF pups with either conventional microbiota or a consortium of *Bifidobacteria*, which dominate the early postnatal microbiome in humans.^42^ However, the mechanism regulating communication between *Bifidobacteria* and cerebellar microglia is still unclear, and might be direct (eg. via circulating metabolites) or could involve other cell types, or indeed could be secondary to effects on the Purkinje neurons themselves. Nonetheless, this study suggests microglia could be critical in linking early-life neuroimmune interactions and neurodevelopment to microbiome composition. Further studies should seek to determine how early gut microbiota influence microglia, and whether this communication axis could affect the genesis of neurodevelopmental disorders.

In adult animals, gut bacteria exert a constitutive influence on microglial physiology via release of short-chain fatty acids (SCFA) (Figure 1). Microglia from adult germ-free animals display a distinctive phenotype characterized by increased density and aberrant morphology, with longer processes and overlapping territories.^43,44^ Intriguingly, the transcriptomic phenotype of germ-free microglia appears to be immature compared to SPF controls, with increased expression of proliferative markers and decreased expression of genes relating to immune function (eg *Cd86, Ly86, Hif1a*) and other markers of mature adult microglia (eg. *Cst7, Neurl3*).^11,43^ It is therefore increasingly appreciated that colonization with gut microbes may provide an environmental cue driving microglia to assume their fully mature phenotype, which occurs naturally around the same time. Moreover, since ablation of the microbiome in SPF animals via broad-spectrum antibiotics largely phenocopies the effect of germ-free housing on microglial phenotype, this instruction clearly occurs constitutively, rendering microglia sensitive to real-time perturbations in gut microbe composition.^43^ Interestingly, colonization of germ-free animals with a defined repertoire of 15 species known as altered Schaedler flora (ASF) was insufficient to reproduce the effect of SPF housing.^43^ This suggests that complex microbiota may be better able to support microglial maturity than other, defined communities. However, it is unclear whether the inability of ASF flora to replicate the effects of SPF housing is truly related to complexity, or rather to insufficient production of particular metabolites such as SCFAs, which might be adequately provided by different species in a similarly defined consortium. Remarkably, it was recently reported that, of the 3 major SCFAs produced by the gut biota (acetate, propionate and butyrate), only acetate is in fact necessary and sufficient for microglial maturity, with neither propionate nor butyrate able to influence the phenotype of germ-free microglia.^44^ Thus, production of acetate alone may be one of the most critical parameters when considering the effects of microbial communities on microglial state.

The immature phenotype of germ-free microglia is not limited to effects on morphology and gene expression in homeostasis. Instead, germ-free microglia also exhibit defective responses to immune stimuli. In SPF mice, intracerebroventricular (i.c) injection of the bacterial endotoxin lipopolysaccharide (LPS) induces a robust TLR4- and NF-κB-dependent response resulting in upregulation of inflammatory mediators including TNF-α, IL-1ß and CCL2. In germ-free animals, this response is markedly diminished, indicating an immunologically immature state.^43^ Similarly, infection with lymphocytic choriomeningitis virus (LCMV) in germ-free animals produces lower induction of *Il1b* and *Tnfa*, as well as the immediate-early genes *Fosb, c-fos* and *c-jun* compared to SPF controls.^43^ Interestingly however, germ-free microglia displayed normal induction of the antiviral signaling molecules *Cxcl10, Irf7* and *Isg15* following LCMV infection.^43^ This could indicate that lack of microbiota induces a specific signaling defect in microglia which impairs certain response pathways but leaves typical antiviral signaling mediated by interferons and RNA sensing pathways intact. The exact nature and mechanism of the signaling alterations induced by lack of microbiota are still unclarified, but could prove critical when drawing mechanistic links between microbiota perturbations and microglial disease responses.

### Mechanisms of microbiota-microglia communication

**Figure 2.**
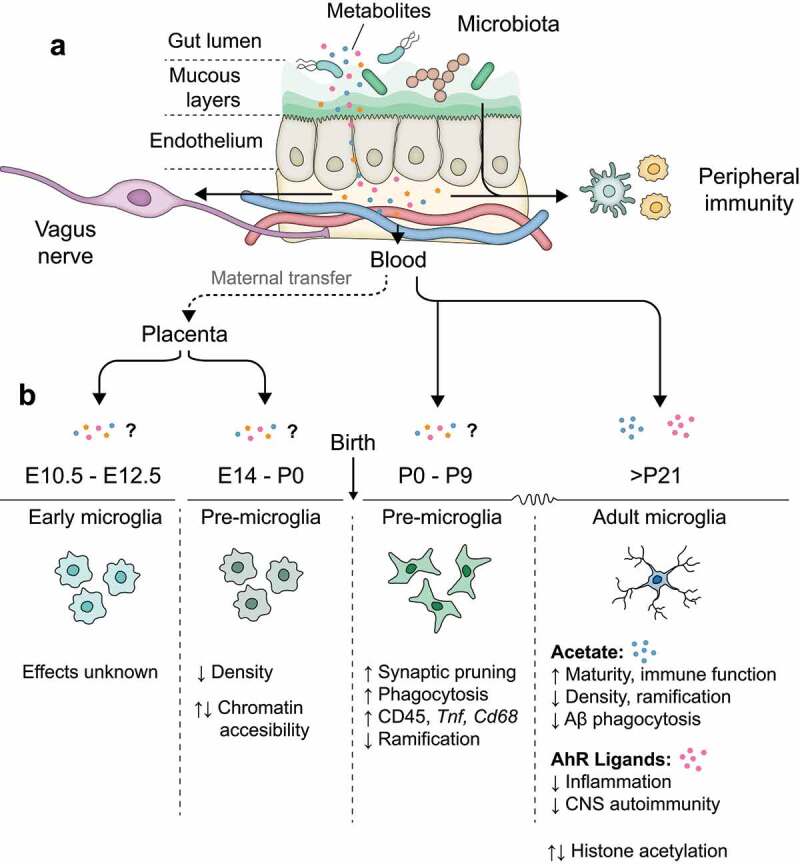
Microbiota-microglia interactions. (a) Microbiota are confined to the gut lumen, separated from the lamina propria by gut endothelial cells. Microbiota-derived metabolites can penetrate the endothelium and enter the bloodstream, as well as influencing local immune cells and vagal afferents. (b) Effects of microbial metabolites on microglial function under steady-state across developmental stages. Evidence suggests that microglial density, epigenetics and transcriptomics are affected by microbiota at all stages of life.

Given the separation between microglia and commensal microbes, interactions are generally only possible via circulatory factors or via altered activity in an intermediate cellfor example, neuronal signaling via the vagus nerve. On the simplest possible level, molecules produced by gut microbes can enter the blood through the intestinal wall, subsequently entering the brain and acting on microglia directly to alter their physiology (Figure 2). However, microglial phenotypes are also sensitive to changes in other CNS cells such as astrocytes, as well as to alterations in peripheral immunity and metabolism. Indeed, relatively few studies have so far demonstrated clear-cut examples of direct microbiota-microglia communication, and in most cases the involvement of additional cell types or signal relays cannot be excluded. Moreover, direct or indirect modulation of microglia by the microbiota can likely occur both in the form of constitutive signaling and epigenetic effects imprinted developmentally. The latter possibility remains somewhat underexplored compared to constitutive signaling pathways, but two studies have so far confirmed differences in chromatin accessibility and histone modification patterns between SPF and germ-free microglia.^12,44^

Currently the best-studied microglia-microbe interactions occur via circulating SCFAs, which are produced by gut bacteria via fiber fermentation. The three most abundant SCFAs produced by the microbiota are acetate, propionate and butyrate, consisting of 2-, 3- and 4-carbon chains, respectively. It was recently discovered that acetate alone is able to reverse many features of the germ-free microglial phenotype, while propionate and butyrate are entirely ineffective.^44^ Thus, while SCFAs had generally been regarded as a single class of metabolite, it appears that their effects on microglia are divergent, with acetate being the primary mediator of microglia-microbiota signaling.^44^ The mechanism by which acetate affects microglial phenotype remains largely unclarified, though several potential pathways have been identified.

Recent data using radiolabelled carbon tracing indicate that gut-derived acetate in the blood is capable of entering the brain and is incorporated into the microglial Krebs cycle.^44^ This same report also linked acetate availability to metabolic alterations in germ-free microglia. Specifically, germ-free microglia exhibit increased mitochondrial mass compared to SPF controls, which is coupled to reduced activity of the mitochondrial electron transport chain complex II.^44^ This defect is rescuable by acetate supplementation and may relate to lack of acetyl Co-A, resulting in increased generation of oxaloacetic acid, a known complex II inhibitor.^44^ Whilst it is unclear whether this metabolic defect is related to the other functional alterations in germ-free microglia, it is feasible given the now widely recognized role of metabolism as a central regulator of macrophage function.^45,46^ These data therefore support a model whereby microbiota modulate microglia by providing a carbon source required for normal metabolism, thus forming a direct link between diet, gut microbiota composition and microglial state. This idea is particularly compelling given that the primary receptor for acetate, FFAR2,^47^ is not expressed in the brain, thus making direct effects of acetate on microglia via GPCR signaling unlikely.^43^

It is especially interesting to consider the effects of acetate on microglial metabolism in the 5xFAD AD model, where germ-free animals exhibit greater microglial phagocytosis and reduced plaque pathology, which is reversible by acetate supplementation.^44,48^ Exposure to Aβ is well described to induce a shift in microglial metabolism toward glycolysis, which over time supports inflammatory polarization and subsequent inhibition of phagocytosis, thus promoting accumulation of amyloid plaques.^49–51^ Indeed, GF microglia exhibit lower induction of glycolytic and proinflammatory genes in the 5xFAD model than their SPF counterparts, which is largely dependent on acetate.^44^ Thus, acetate availability appears to constitutively modulate both dependence on glycolysis and inflammation in amyloid-exposed microglia, which may result in phagocytic suppression and increased plaque accumulation. However, exactly why this occurs remains unclarified.

Additional explanations for the maturation-inducing effect of acetate on microglia include protein acetylation and activation of GPCR signaling pathways. Indeed, microglia in mice lacking the SCFA receptor FFAR2 exhibit similar defects to those from germ-free animals despite a lack of FFAR2 expression in the brain, indicating that microglia could be affected by SCFA signaling in other organs.^43^ However, supplementation of germ-free animals with propionate, another FFAR2 agonist, does not affect microglial phenotype, with acetate being the only SCFA capable of reproducing microbiota-induced microglial maturation. As such, it is possible that FFAR2 is dispensable for the effect of acetate on microglia, but its deletion phenocopies acetate deprivation via a separate, unclarified mechanism.

Protein acetylation constitutes another route by which acetate availability might directly influence microglial physiology. Acetylation is an extremely common post-translational modification with profound consequences for the function of various immunological proteins.^52^ For example, the transcription factor NF-κB, which is critical for the generation of inflammatory responses, relies on acetylation of the p50 and p65 subunits for DNA binding and full transcriptional activity, respectively.^53,54^ LPS elicits classical NFκB-dependent responses which are blunted severely in germ-free microglia. This raises the possibility that the dimished LPS responsivity observed in the absence of microbiota could be due to lower acetate availability leading to insufficient acetylation of NF-κB or other immunologically important proteins. However, to date no studies have examined the effect of microbial acetate on acetylation of the general microglial proteome.

Another facet to protein acetylation involves epigenetic modification of histone proteins by histone acetyltransferases (HATs). Acetylated histones generally result in more accessible chromatin and increased gene transcription,^55^ and are a key mechanism regulating immune cell effector functions following stimulation.^56,57^ Acetate supplementation produces increased histone acetylation in various organs,^58^ including the brain,^59^ and microbiota have been reported to regulate patterns of acetylation across a variety of histone sites via release of SCFAs.^58^ Gut microbiota-derived acetate may affect histone acetylation by increasing the availability of acetyl-CoA, a necessary substrate for HAT activity, or via inhibition of histone deacetylases (HDACs).^59^ In microglia, gut microbiota have been shown to regulate acetylation at the 9^th^ lysine of histone 3 (H3K9),^44^ a modification associated with increased promoter activity. Whilst levels of H3K9 acetylation appear to decrease overall under GF conditions, many genes also display increased H3K9 acetylation compared to SPF microglia.^44^ This is in agreement with a previous report which identified differentially accessible chromatin regions between GF and SPF microglia via ATAC-Seq beginning in early development.^12^ Thus, the microbiota clearly regulate microglial histone acetylation, which could underly some aspects of their effect on microglial function. However, whether or not this effect can be attributed to acetate is unclear, since propionate and butyrate are also known HDAC inhibitors capable of influencing epigenetic state^60–62^ and the effect of individual SCFAs on microglial histone modification has not been assessed. Moreover, it is unknown whether microglial histone modification patterns are controlled dynamically by the microbiota or are imprinted during development and remain static thereafter, as was recently suggested for the liver.^63^ Finally, it would be helpful to dissect precisely which aspects of the germ-free microglial phenotype are dependent on epigenetic modifications, and whether this could account for the differing effects of antibiotic treatment in SPF animals versus acetate supplementation in GF conditions.^43,44,48^

Taken together, the evidence for a direct microbiota-microglia communication axis mediated by acetate is compelling, but key questions remain around the precise nature of the effect, which may involve multiple parallel functions of acetate as both a signaling molecule and metabolic substrate. A major question moving forward is what role other microbial metabolites might play in instructing microglial phenotype. Acetate supplementation reverses most of the metabolic and phenotypic differences between GF and SPF microglia, but does not completely normalize the transcriptome under either homeostatic or pathological conditions.^44^ This could be explained by lingering epigenetic differences between GF and SPF animals, and/or could imply the presence of extra signaling factors which regulate microglial phenotype more subtly. Aryl hydrocarbon receptor (AhR) ligands have been demonstrated to influence microglial state during CNS autoimmunity,^64^ but their effects under homeostatic conditions have not been reported. Microglia are also affected by microbial products during embryonic and postnatal development, but the molecular players are entirely unknown. Acetate has been shown to be differentially abundant in SPF versus germ-free embryos, along with a variety of compounds including the AhR ligand indoxyl-3-sulfate, trimethylamine-N-oxide (TMAO), hippurate, N, N, N-trimethyl-5-aminovalerate (TMAV) and imidazole propionate.^65–67^ One recent study identified defective thalamocortical axon development in germ-free embryos, which is rescued by maternal supplementation with the latter 4 compounds listed above, but not by SCFAs including acetate. Whether embryonic and early postnatal microglia could also be regulated by these compounds, acetate, or AhR ligands would provide an interesting basis for future studies.

### Microglia-microbiome communication in disease

Microglia are active regulators of a variety of CNS pathological states, including neurodegenerative diseases such as Alzheimer’s disease and Parkinson’s disease, as well as neuroinflammatory and neuropsychiatric conditions.^2^ Signaling from the microbiota to microglia has therefore been suggested as an environmental factor capable of promoting or ameliorating disease. In support of this idea, microbiota-dependent influences on microglial states have been reported to exhibit a high degree of sex-dependence,^12^ which could help account for the marked differences in susceptibility to certain CNS diseases observed between men and women. A vast quantity of literature has in recent years been generated suggesting roles for microbiome dysbiosis in the pathology of numerous neurological conditions. Since this is too extensive to review in full, we focus here on critically reviewing the most compelling evidence linking communication between microbiota and microglia to neurological disorders.

#### Alzheimer’s disease

Alzheimer’s disease (AD) is a chronic neurodegenerative disease characterized by progressive brain atrophy and profound memory loss, which is accompanied by deposition of amyloid plaques and overabundance of hyperphosphorylated tau protein.^68^ Amyloid plaques are composed of high-order aggregates of amyloid-beta (Aβ), a peptide produced by cleavage of amyloid precursor protein (APP).^69^ While the pathology of AD is complex and incompletely understood, excessive accumulation of Aβ is thought to be a major trigger of subsequent disease processes such as tau hyperphosphorylation, synaptic dysfunction and neuronal loss.^69–71^

The microbiota are proposed to play a major role in AD, which could occur at least partially via microglia-mediated mechanisms. Several studies have reported decreased richness and diversity of fecal microbiota in AD patients compared with healthy controls.^72,73^ More specific differences on the level of individual phyla and genera have been reported, but in some cases are inconsistent between different studies^72–77^ (reviewed by^78^). For instance, Vogt et al.^72^ noted decreased abundance of Actinobacteria in AD patients, whereas Ling et al.^73^ observed an increase. Similarly, increased abundance of Bacteroides is noted by some, but not other studies.^72,73,77^ One relatively consistent point, however, is that the abundance of Firmicutes appears to be generally decreased in AD patients.^72–77^ Additionally, some functional relationships have been proposed between AD pathology and the balance of inflammatory versus anti-inflammatory species such as *Eschieria/Shigella, E. rectalis* and *F. prauznitii*.^74,75^ While it is entirely feasible that changes in gut microbiota aggravate or promote AD, and evidence exists for altered microbiome composition in AD patients, human clinical studies have thus far been entirely correlative. Moreover, since AD-associated pathological changes begin decades prior to the onset of clinical symptoms,^70^ the most relevant changes in gut biota may be missed by studies focusing on patients with diagnosed AD, and future studies tracking temporal changes in microbiome composition prior to AD onset may be more informative.

Whilst mechanistic associations between gut microbiota variation and AD pathology are difficult to obtain in humans, there is now a wealth of corroborating data confirming that mouse models of AD are extremely sensitive to changes in gut bacteria. The first study to address this issue demonstrated that antibiotic administration to APP/PS1 mice (high-dose for one week followed by chronic low-dose) reduced amyloid plaque burden.^79^ Subsequent studies have validated that germ-free housing or antibiotic treatment reduce plaque pathology in the APP_Swe_/PS1_ΔE9_,^80,81^ APPPS1-21^81–83^ and 5xFAD^44,48^ models. Mechanistically, this effect seems to be independent of amyloid processing pathways,^48^ and instead reflects altered microglial activity.^44,48^ Microglia from germ-free animals exhibit greater induction of phagocytic genes such as *Axl, Apoe* and *Clec7a*, as well as higher expression of reactivity markers such as *Cst7* compared to SPF controls.^44,48,84^ This is coupled to increased levels of amyloid phagocytosis in germ-free microglia, indicating that microbiota interactions direct critical microglial functions in AD models.^44,48^ Interestingly, recent studies report that, similarly to the steady-state phenotypic differences observed in germ-free mice, the effect of microbiota on amyloid pathology appears to be mediated primarily SCFAs, specifically acetate.^44,84^ Thus, clear evidence exists for an acetate-mediated microbiota-microglia signaling axis which promotes amyloid accumulation in AD mouse models. Most intriguingly, a recent study in human AD patients reported that amyloid burden correlates positively with serum acetate levels, but negatively with serum butyrate and propionate.^85^ This supports a model whereby acetate may act via microglia to promote amyloid pathology in AD, whereas butyrate and propionate are unable to modulate microglial physiology and instead are protective due to effects on other cell types. While causative associations between individual microbes or metabolites and human AD remain unproven, these results suggest that AD interventions targeting gut-derived SCFAs should pay close attention to the role of acetate versus propionate and butyrate in modulating amyloid burden.

#### Parkinson’s disease

Parkinson’s disease (PD) is a complex neuropathology involving degeneration of dopaminergic neurons in various brain regions such as the substantia nigra, along with extensive intracellular accumulation of aggregated α-synuclein protein.^86^ Symptoms include progressive motor dysfunction along with cognitive impairment, depression, and sleep disruption.

Gut-brain signaling has long been suggested to be of particular relevance for PD, especially considering that α-synuclein pathology is often observed in the gut,^87^ and that gut inflammation and motility problems are common in PD patients. It has even been postulated that α-synuclein aggregates may in some cases originate in the gut and propagate to the brain in a prion-like manner via the vagus nerve, though this idea remains controversial.^87–89^ A role for the microbiota in regulating PD pathology could therefore be multifaceted, influencing the local generation of toxic α-synuclein species or distantly affecting microglial phenotypes in affected regions of the CNS, as occurs in AD models. Some studies have demonstrated altered gut microbiota composition in PD patients, though the results are likely affected by drug treatment and, as with AD, it is unclear whether gut flora changes precede brain pathology.^90^

To date, the only direct evidence for involvement of the gut microbiota in PD comes from rodent studies. In an α-synuclein overexpression (ASO) model, GF mice were protected against gut motility defects, microglial reactivity, brain inflammation and ultimately motor deficits.^91^ Similarly to in AD models, the detrimental effects of complex gut biota on neuropathology in ASO mice are reproducible by supplementation with SCFAs.^91^ Interestingly, colonization of GF ASO mice with microbiota samples from PD patients generally (but not invariably) produced greater motor impairment than with samples from healthy controls.^91^ However, it is unknown whether this effect is due to differences in SCFA production between PD-derived and control microbiota or whether other mechanisms are responsible.

Most studies assessing SCFA levels in PD patients have found decreased concentrations in fecal samples, suggesting dampened SCFA production.^92–95^ However, it has also been suggested that, due to the frequent alterations in gut function accompanying PD, stool SCFA measurements may not accurately represent systemic exposure compared to healthy controls.^95^ Measurements of circulating SCFAs in serum are less common, but have been variously reported as decreased,^96^ increased,^95^ or unchanged^97^ in PD patients. Moreover, correlations between individual SCFAs and clinical symptoms are most often reported for propionate and butyrate,^95,97^ which in mice do not directly affect microglia.^44^ Thus, there is currently little evidence to support acetate-mediated microbiota-microglia signaling as a causative mechanism in PD patients. While this does not rule out a role for the microbiota in modulating microglial phenotype in human PD, it may be that other metabolites are responsible,^98^ or indeed a more complex mechanism such as via systemic immunity, gut barrier integrity or neuronal signaling. Further mechanistic studies in mouse models may prove useful to elucidate exactly why PD patient-derived microbiota elicit greater neurodegeneration in mouse models, and whether microglia are critically involved.

#### Multiple Sclerosis

Multiple Sclerosis (MS) is an autoimmune condition generally caused by autoreactive immune responses directed against myelin components, resulting in the formation of brain lesions exhibiting prominent inflammation, demyelination and blood–brain barrier disruption.^99^ While the disease course of MS is highly variable, most patients eventually experience irreversible neurological damage, impairment and disability which continues to worsen over time. The causes of MS are unknown, though previous infection with Epstein-Barr virus (EBV) appears near-universal amongst MS patients, and may promote the generation of cross-reactive T cells via molecular mimicry.^100^ As a primarily immune-mediated condition, immunomodulatory therapies in MS have shown some clinical success in reducing relapse frequency and slowing disease course.^99^

Multiple states of reactive microglia can be observed in MS, and likely play multifaceted roles in regulating the progression of pathology.^34,101^ One the one hand, microglia can contribute to the intense inflammation typical of MS lesions, inducing neuronal damage and demyelination.^101^ On the other hand, microglia are also critical for phagocytosis of myelin debris and stimulating oligodendrocyte differentiation and remyelination.^101^ Thus, microglia constitute a key target cell for any interventions aimed at modifying the course of MS. Unlike typical neurodegenerative conditions, the peripheral immune system is heavily involved in MS, and overall pathology is influenced by the properties of both CNS-resident cells and infiltrating peripheral cells. Thus, whilst there is evidence that gut bacteria are capable of modifying MS pathology, it must be considered that their effects can be mediated not only through changes in microglia but also in peripheral immunity. Indeed, a variety of studies have demonstrated that gut flora are required for the generation of autoreactive T-cell responses in experimental autoimmune encephalomyelitis (EAE), a commonly used model of MS.^102,103^ Thus, germ-free animals are generally resistant to EAE pathology due to defective T-cell differentiation. In humans, differences in the ability of gut bacteria to stimulate inflammatory versus regulatory T cell responses may constitute a key environmental factor determining MS risk.^104^ It is also for this reason that GF animals are of limited use for studying microbiota-microglia signaling in EAE, and therefore only few studies have successfully demonstrated mechanistic links between gut biota and microglial phenotypes in EAE models.

One key study examining microglia-microbiota crosstalk in EAE did so by focusing on the aryl hydrocarbon receptor (AhR), a major mediator of gut-immune signaling.^64^ Mice harboring AhR deletion in microglia following transient CX3CR1-CreERT2-mediated recombination exhibit exacerbated clinical scores after immunization with MOG peptide.^64^ This was found to be due to reduced AhR-dependent secretion of TGF-α and increased production of VEGF-B from microglia, causing increased pro-inflammatory astrocyte polarization. AhR ligands are produced by the gut microbiota via tryptophan metabolism, and restriction of dietary tryptophan in WT mice phenocopied the effect of microglial AhR deletion.^64^ Conversely, supplementing a tryptophan-deficient diet with AhR ligands such as indole-3-carbinole (I3C) rescued the worsened EAE pathology in WT, but not microglial AhR-deficient mice.^64^ Thus, gut flora are capable of influencing microglial phenotypes in EAE independently of changes in T cell-mediated immunity, and may be critical in transducing diet-related environmental variables to EAE risk. These results build on a previous study indicating that AhR ligands can also signal via astrocytes, and that AhR signaling may form part of the protective influence of interferon-beta in MS.^105^ Thus, microbial tryptophan metabolism and AhR ligands are a promising lead for harnessing endogenous microglia-microbial signaling pathways in MS, and efforts to validate their role in human subjects are ongoing.^106^

#### Autism spectrum disorder

Autism Spectrum Disorder (ASD) comprises a group of related neurodevelopmental disorders whose manifestations vary widely in both type and severity but can include learning and social impairment as well as repetitive behaviors.^107^ The etiology of ASD is poorly understood, but is generally thought to be a multi-hit process involving genetic predisposition in combination with environmental triggers during critical developmental periods.^108^ Gastrointestinal symptoms are common in individuals with ASD, suggesting a link between gut function and ASD symptoms.^109^ The gut microbiota is a prime example of an environmental factor which is established during early development and proceeds to regulate both brain and gut physiology, and is often dysregulated in ASD.^1^ There is currently intense interest in microbiota-based therapies for ASD, some of which have already shown promising results in initial trials.^110^

Microglia are presumed to be involved in ASD pathology due to their intimate contribution to neurodevelopment and ability to regulate brain function through release of inflammatory mediators and neurotrophic molecules.^111^ Indeed, some degree of microglial abnormalities and brain pathology have been noted in ASD patients.^112–116^ However, altered microglial states could occur downstream of neuronal abnormalities in ASD and definitive causal associations are still lacking.

Currently some of the best evidence for causative roles of microglia and microbiota in ASD come from models of maternal immune activation (MIA). Viral and bacterial infections during pregnancy are a known risk factor for the development of ASD in humans,^117,118^ and offspring of mice injected with the viral mimetic poly(I:C) during pregnancy exhibit a phenotype which shares some features with ASD, including social interaction deficits and increased repetitive-like behaviors.^119^ This effect depends on induction of an acute inflammatory response in the maternal serum, including IL-6 and IL-17a.^120–122^ Maternal cytokine elevation results in subsequent production of inflammatory mediators in the fetal brain,^123^ and increased concentrations of some cytokines can be detected during postnatal development,^124^ though precisely which mediators are altered at which time points vary between studies.^125^ Microglia from MIA offspring display an altered phenotype characterized by premature induction of the mature gene signature in neonatal pups, as well as decreased phagocytosis and higher basal expression of inflammatory genes in adulthood.^11,126^ While these results in combination with human data suggest that microglial dysfunction can promote ASD pathogenesis, the exact contribution of microglia to cognitive and behavioral outcomes is still unclear, and many studies report conflicting results with regard to microglial reactivity following MIA.^127^

MIA pathogenesis is also strongly linked to gut microbiota. Segmented filamentous bacteria (SFB) in the maternal gut are required for induction of the IL-17a response following poly(I:C) exposure, and mice lacking SFB do not show the expected behavioral deficits in MIA offspring.^122^ Thus, the ability of viral infection to promote ASD may depend on the presence of commensals which direct maternal immune responses. Gut bacteria may also have constitutive effects on ASD pathology, since colonization of GF mice with gut biota derived from individuals with ASD promoted behavioral symptoms typically observed in ASD mouse models to a greater extent than that from neurotypical controls.^128^ This effect has been ascribed to differential abundance of microbial metabolites, which is commonly observed in ASD,^129^ leading to altered expression of ASD-linked genes in the brain.^128^ Conversely, treatment of ASD model mice with normal human commensals such as *B. fragilis* has been shown to reduce the severity of behavioral symptoms.^130^ These studies argue in favor of a causative role of microbiota alterations in ASD pathology, both in regulating predisposition via maternal immune activation and in promoting behavioral symptoms in later development, acting as an environmental “hit” in concert with other environmental and genetic factors. Given the abundant microbial metabolite alterations in ASD patients,^129^ it is feasible that their effects are mediated at least partially via alterations in microglial activity, though to date no studies have confirmed this.

In contrast to this idea, one recent study has questioned the causality between gut microbiota composition and ASD, suggesting instead that the behavioral symptoms of ASD drive microbiota alterations via less varied food intake.^131^ Moreover, most studies to date investigating treatment of ASD patients with pre- or probiotic formulations have been described as either inconclusive or unreliable by a recent meta-analysis.^110^ Despite this, the first trial of fecal microbiota transfer in ASD has reported promising outcomes.^132,133^ Thus, the role of both the microbiota and microglia as targets for ASD are still somewhat controversial, but remain an active and promising area of research.

## Outlook and future challenges

To date, research into microglial modulation by microbiota has uncovered several promising phenomena of potential relevance to neurological disorders. However, the field is still in its infancy and significant challenges in the interpretation and translation of current findings into disease settings remain to be addressed. The first major caveat to microbiome research in general is that studies in humans largely produce correlative data with little mechanistic insight, and establishing causality between human diseases and microbiome alterations is extremely difficult. This is particularly true of diseases such as AD which exhibit long periods of clinical latency before symptoms manifest. It is for this reason that mouse models are generally relied upon for mechanistic studies seeking to dissect the contribution of microbiota perturbations to disease. However, translating mouse findings back into humans has a notoriously low success rate, which has been attributed not only to issues in preclinical study design, but also to more fundamental flaws in the accuracy with which human diseases are modeled in mice.

Germ-free mice, for instance, have provided invaluable insights into the essential role of gut microbes in mammalian biology, but are clearly highly unphysiological and unsuited to modeling the effects of subtle changes to a complex microbiota such as those observed in humans. Many additional gnotobiotic mouse models exist, harboring varying numbers of known bacterial strains, though other than the ASF model their effects on microglial function remain uncharacterized. These models could provide a useful reductionist approach to identify particular bacterial strains which might promote microglial function, either via acetate release or other mechanisms. However, these suffer the same drawback of questionable translational relevance.

Theoretically, this limitation can be overcome by use of fecal microbiota derived from human subjects. However, these models should also be interpreted with caution due to various issues including incomplete colonization of the recipient animal and the absence of additional relevant host and environmental factors.^134,135^ It is also increasingly appreciated that even SPF mice have unnaturally sparse microbiota lacking many natural symbionts and pathogens, which results in relatively immature immune system highly dissimilar to that of humans.^136–139^ Interestingly, colonization of laboratory mouse strains with complex “wild” biota or infection with pathogens results in a much closer approximation of human immunity than can be achieved in SPF animals.^136–139^ However, it is currently unclear whether microglial function is affected by colonization with wild biota, or whether its effects relate purely to microbial diversity or rather to the presence of specific immunostimulatory microbes absent from SPF mice. Nonetheless, conventional SPF mice clearly suffer from a paucity of microbial stimulation at baseline, which could render investigation of microbiota-immune interactions prone to artifacts.

Thus, to maximize the impact of microbiota-based therapies on human medicine it is important to establish robust causal relationships in human-relevant models. It would be intriguing for future efforts to establish whether the presence of wild-like microbiota affects microglial function, since this could provide a more robust platform for investigating human-relevant pathways of microbiota-microglia communication and increase the translational success of any resulting strategies. It is equally critical for future studies in human patients to gather high-quality data combining microbiota profiling with metabolomic analyses, ideally including longitudinal study designs and diverse patient cohorts to maximize mechanistic interpretability and identify high-confidence targets for animal model validation. It should also be appreciated that, although direct signaling between microbiota and microglia is a compelling concept, optimal clinical success will likely only come by targeting multiple systems including gut and metabolic function, as well as peripheral immunity.

Currently, the only robustly defined routes of direct communication between microbiota and microglia occur via acetate and AhR ligands, the latter of which has only been demonstrated in the context of EAE.^64^ Given the great diversity of microbially derived molecules permeating the body, it seems likely that at least some are capable of modulating microglia in ways which remain to be discovered, especially considering that the bulk of studies to date have been performed in a single strain of laboratory mouse under conditions of artificially low microbial diversity. It would therefore be interesting for future studies to dissect the effect of both wild mouse microbiota and transplanted human microbiota on microglial phenotype, with particular focus on the underlying metabolites and mechanisms. Extra care should also be given to examining the relationship between microbiota and microglia at different developmental stages. Embryonic, early postnatal and adult microglia are not only intrinsically different, but also very likely experience divergent sets of microbial cues. Thus, the existence of developmentally specific signaling axes should be considered. Another currently overlooked facet to microbiota research is the role of non-bacterial species such as fungi. Fungi have already been demonstrated to promote social behavior via IL-17 signaling in neurons,^140^ and to modify Alzheimer’s pathology in APP/PS1 mice via an unclarified pathway.^141^ Thus, fungi may provide additional routes of microglial modulation by microbiota and are deserving of further study in both humans and mice.
